# Pichi in the Diseases of the Genito-Urinary Tract

**Published:** 1893-11

**Authors:** 


PICHI IN DISEASES OF THE GENITO-URINARY TRACT.—Friəndlaender (*Therap. Monatshefte*, July 18, 1893) speaks very favorably of the action of pichi in cystitis, prostatitis, acute gonorrhea, vesical neuroses and epididymitis. He administers the fluid extract in the dose of a teaspoonful thrice daily. Gastric disturbance, cutaneous eruptions and pain over the loins were not noticed after the administration of the drug, as is often the case after the use of the balsam of copaiba, or oil of sandal wood. The following six cases are recorded: (1) A man, aged 52 years, with nervous irritability of the bladder lasting two weeks. The symptons disappeared six days after the administration of pichi. (2) A man, aged 21 years, with edema of the prepuce, acute gonorrhea and acute cystitis. In five days the acute symptoms—frequent micturition, dysuria and swelling—had almost completely disappeared. (3) A man, aged 58 years, with prostatic hypertrophy and chronic cystitis. In three days the urine became clear and of acid reaction and the obstruction was lessened. (4) A man, aged 23 years, with pain in the sacral region from chronic prostatitis. In eight days the pain had entirely disappeared. (5) A man, aged 21 years, with acute gonorrhea. In four days the discharge hac decidedly lessened and the pain and swelling almost entirely disappeared. (6) A man, aged 32 years, with orchitis, epididymitis and subacute gonorrhea. He was ordered a suspensory and fluid extract of pichi. In a week the pain over the sacrum was lessened, the epididymis was reduced one-half and was almost painless, and the appetite and general health were greatly improved, In conclusion the writer believes that pichi is a valuable addition to the therapeutics of urinary diseases, and from its lack of pernicious qualities is superior to balsam of copaiba, oil of sandal wood and turpentine.—*Ex.*

